# “Diphtheria and Bacteria:” a Criticism Criticised

**Published:** 1881-10

**Authors:** Rollin R. Gregg

**Affiliations:** Buffalo, N. Y.


### "DIPHTHERIA AND BACTERIA."

### A Critic Criticised.

Rollin R. Gregg, M. D., Buffalo, N. Y.

*Editor of* "The Homœopathic Physician:"

I have read, re-read and read again, in the June, 1881, number of your journal, Dr. Wells' twelve-page criticism of my previously published paper upon "Diphtheria and Bacteria," and to say I am surprised at it, would but feebly express my emotions. I am amazed that a physician of Dr. Wells' character and standing, should deliberately proceed to raise false inferences against any member of the profession, and then arraign, try and condemn him upon such inferences; I am amazed that a man of his years should display such anxiety to seek and impute unworthy motives to one who never did him harm; and I am amazed that one of his conceded talents, in other fields, should so foolishly think I had written such a paper as the one he criticises, and left myself as illy prepared to defend, as he has shown himself to criticise it. But, before entering upon a defense of that paper, a little personal explanation seems not out of place.

If I have had any patron saints in medicine, besides Hahnemann and Bœnninghausen, Dr. Wells has taken a high rank among them. During the twenty-eight years that I have practiced homœopathy, I have often reflected with pride that he belonged to our school, and that such men honored it. I have always read, and with pleasure, all the articles from his pen bearing upon therapeutics, that I have ever seen, and have repeatedly gone back to them under trying emergencies to sustain me in what I believed to be the right thing to do in the treatment of the gravest forms of disease. This I have even done within the last year, and shall do it again and again in the future, no doubt, upon all occasions when I feel the need of such support.

True, I never expressed as much to Dr. Wells personally or by letter, not having had the pleasure of a personal acquaintance with him, nor have I ever said as much in public print; but that furnishes him no excuse for unjustly assailing any member of the profession, and thereby destroying the high esteem in which that member may have held him. And let this be a warning to him and other prominent physicians in the future, to be more chary of their unfair criticisms of others' ideas that they do not understand, and when they are themselves in no wise assailed. If they are not, they may, as in this case, needlessly and cruelly wound the best of personal sentiments, and run the risk of turning high admiration into bitterness and rancor—only in this case there will be none of the latter, after I once balance accounts with the doctor, and providing he demeans himself henceforth in a way that will at all permit of the continuance of my high regard for his eminent talents in other departments of medicine. But let us pass on to much more important matters.

The very first word with which Dr. Wells starts out in his criticism of my views, namely, the word "hypothesis," is false, utterly false,* as applied to my investigations and conclusions in any department of pathology, as I will now proceed to show, in part by his own language, and in part by a truthful and candid statement of facts. He says:

> "It has always been easier to imagine how a matter may be, or may have been, than to find out how it is, or was, by careful collation and examination of facts, and from that construct a whole of truth, based on this only sure foundation."

Dr. Wells' "imagination" to the contrary notwithstanding, I have builded *securely* and *permanently* upon his "only sure foundation," as, I think, even he will see by the following record of the great care taken in all my pathological investigations:

Twenty-seven years ago last summer, and in a quite accidental

* Let no one think that I here use this word "false" in its offensive, personal sense; but simply because it is the only word that fitly characterizes the great error Dr. Wells has fallen into in applying the word "hypothesis" to my deductions.

manner, that is, through a careful investigation of the morbid condition of a case of incipient phthisis, but without the least suspicion as to what I was to find that was new, I fell upon the first great fact that aroused me to what, it seems to me may be said, without boasting, has been one of the longest, most thorough and most exhaustive researches into the cause and nature of tubercle ever given to that subject by any individual member of the medical profession. Scarcely a day in all the time since, has the subject been wholly out of mind, and for years I knew not with certainty where I was drifting, to what haven my investigations would finally lead me. Established one theory which, though a help to me by pointing in the right direction, had to be abandoned because of its error in part and its insufficiency in covering the whole ground; and had to discard many other minor conclusions reached during the first seven years, in the absence of a full and complete knowledge of the whole subject.

One year subsequently to the time just named, or twenty-six years ago, I stumbled as accidentally, or, at least, through an unexpected result in the successful treatment of a consumptive subject, upon another fact, entirely disconnected in my mind at the time from the other, but scarcely less important in its far-reaching results. Then, in each of the succeeding six years, a fragment was here and there secured that fitted one or the other of the above-named fragmentary facts, until September 21st, 1861, the key was found which unlocked the whole inner temple of this complicated subject, and showed the exact position and relations to each other of all the previously found, and many more fragmentary facts.

Well, it was while studying and familiarizing my mind with what this key unlocked and exposed to view, and entirely in its application to tuberculosis, that I first, and just as accidentally, fell upon the evidences, which seemed conclusive, that the profession was greatly at fault in its views of the ætiology of diphtheria, and of much of its pathology as well. Thus it will be seen that originally I had no intention, as I had none, of investigating diphtheria, much less of writing upon it, and only did so as the result of an accident, as it were. But having my attention called to the subject so pointedly as it was, and the more I investigated, the more plausible the new truths seemed, it appeared like a neglect of duty not to give them some heed. Hence, during all the twenty years since, I have only given those truths less attention than tuberculosis.

To show the care given to details in my researches, let me relate an incident.

Through four successive years, or from 1861 to 1865, I gave almost every spare moment I could get from professional duties during the day, and a greater or less portion of half the nights in all that time to a most careful study into the cause and nature of tubercle; and especially into the origin of tuberculous corpuscles, to learn, among other things, if there could be any possible doubt that these were *only* decolorized and shriveled blood-corpuscles, as all the new facts seemed to show. Then, at a great pecuniary sacrifice, left my business for several months that my whole time might be given up to that study. Well, after several weeks of this unmolested study, I awoke one morning before sunrise with this thought:

"The cartilages have no capillary or other blood-vessels entering them, and, therefore, if tubercles have ever been found in cartilages, then all the conclusions and results, in a specific sense, at least, of all my four years of intense and wearying study were overturned — dashed to the ground in an instant. If right that tuberculous corpuscles were only decolorized blood-corpuscles, then tubercles could *only* be found where capillary blood-vessels existed, and there being none of the latter in cartilages, and if tubercles had ever been found in them, then, of necessity, tuberculous corpuscles could not be changed blood-corpuscles, as it would be impossible for these, under any circumstances, to enter the cartilages and be there deposited to make tubercles."

I arose at once, dressed hastily, and commenced a search through all the authorities then at my command, for the proof that should confirm or destroy all my hopes in that direction. Ate little, and scarcely breathed naturally all day, but went on vigorously with my work until late in the evening, when, in the body of a paragraph, in a work upon pathological anatomy, and without a word in the index or contents of the book, or in the heading of the section, to direct me to it, I found this short but welcome sentence: "We do not find tubercle in cartilages." And several months subsequently, found the same fact confirmed by Rokitansky, in this language as nearly as I can give it from memory: "Tubercles have never been found in cartilages."

It has been in this spirit, and with this care that, for *twenty years*, I have investigated the cause and nature of diphtheria as well as of tuberculosis; only, as already said, the former less intently than the latter, until the last few years I have given diphtheria as much or more attention. And now, after all this, to be told by one whose every paragraph of criticism shows conclusively that he has not given as many weeks as I have of years to the earnest, scientific study of the subject, and whose own sad want of knowledge of his theme crops out on every page—to be told, I repeat, in almost every paragraph, and in, what appears to me, far from a gentlemanly manner, that in my labors, "The essential difference between fact and fancy becomes to him, so far as his pet is concerned, a matter beyond his grasp;" that my work "is born so largely of the imagination of its author as to serve well the occasion of a protest;" that it is "giving fancy for fact;" that it is all nothing "but a figment of Dr. G.'s imagination, pure and simple—that it is just this, and nothing more;" that a fact that has been known to every educated physician for a century, "stands wholly on the *ipse dixit* of Dr. G.," etc., etc.; is, to say the least of it, not a very fair recognition of twenty years of hard work, and is very poor encouragement for earnest labor in any field of science.

The love of nature's truths, doctor, is what sustains men in the face of such shameful assaults as these, and that is about the only thing that earnest, truth-seeking men do find in this world to sustain them, when advocating new truths.

You call for proof upon several points, doctor. Of what earthly use would proof have been to you in the frame of mind you were in when you wrote that criticism? Greatly to your discredit, you totally neglected to consider proof that I did give upon three of the most prominent of all points in diphtheria, and from three among the most prominent of all authors; and by giving which I exceeded the limits originally assigned me in the journal where it first appeared. And here, doctor, comes up a point on which, it seems to me, you did yourself no little dishonor.

In the paper you criticised, I said distinctly: "The limits of a journal article like this will not allow of one-twentieth the proof being given there is to sustain the views here presented;" and then referred to where much of that proof may be found (in my lately published work); but it would seem that you never looked into that work to find and consider its proof, but purposely restricted your criticism to that short paper, and even ignored the proof in that. If you really want proof, you will find ten or more times as much in my book as in that paper, and I could give you ten or more times as much as in my book, if a purpose could be served by it that would compensate for the labor of writing it out.

But let us look at a little proof, now, upon one point, which it is to be hoped you will not wholly neglect, as you did what was under your eye before. After quoting from me only part of the following sentence: "Fibrin is in excess in the blood in diphtheria, as it is in every other inflammatory disease;" you say "This stands wholly on the *ipse dixit* of Dr. G."

Doctor, in this effort to discredit me, you would unwittingly do me altogether too much honor. I had no hand or lot in establishing that great *fact* in all inflammatory diseases. It was done a century or more ago; or, at least, before I had an existence in this world; and can it be possible that a physician of your intelligence did not know it? I shall have to say to you, as I did to another captious critic last winter on the same point:—

"Shades of Broussais and the 'buffy-coat,' is there even a first-course medical student in the country who does not know that fibrin is *always* in excess in the blood in every inflammatory disease; and is it possible that it can be necessary for the medical profession to go on another century as it did the latter part of the last and first of this century, bleeding patients to death by the hundreds of thousands to try and get rid of said excess in inflammatory blood, in order to prove to some minds that fibrin is in excess in such blood?"

As it seems so necessary, however, to have this fact *re*-established for some minds, will you please consider the following *proof* of it from "Watson's Practice of Physic," page 105:

"In nearly all the strongly-developed acute inflammations, there is an excess of fibrin and of the colorless or lymph globules in the blood. From three parts in a thousand, which, according to Andral, is the average in health, the fibrin has been found to rise to six or eight parts. In some cases, MM. Andral and Gavarret found it as high as ten parts in the thousand; namely, in pneumonia and acute articular rheumatism. The excess of fibrin was noticed by Andral in cellular inflammation, or simple plegmon, in acute inflammations of the skin, as in burns and erysipelas, in mercurial stomatitis, in phlegmasiæ of the mucous membrane of the respiratory and digestive organs, in acute cystitis, either simple or combined with nephritis, in all the plegmasiæ of the serous membrane, in inflammation of the lymphatic glands. * * * The increase of fibrin in the blood is manifested so soon as the inflammation begins. * * * It would appear very certain that the formation of the buffy coat in inflammatory diseases is in a great degree dependent upon the excess of fibrin. It is found only in cases where the proportion of fibrin is abnormally augmented."

Thus you may see and rest assured, doctor, that I do not make statements without the best of authority, or the best of reasons, for making them; and you may be just as certain that I lay no claims to original investigations, or discoveries, unless entitled to them. In making the statement, that fibrin is always in excess in the blood in inflammatory diseases, I knew that educated physicians would know the fact and its source, without my offering proof upon the subject. And in conclusion of this point, allow me to say that your utterances upon this question furnish the most powerful plea I have ever seen, for our school giving more attention to pathology than some of its members do; not, however, for the purpose of elevating pathology to usurp the field of therapeutics; but to enlighten us all as physicians, and to broaden our minds in scientific thought.

Now, doctor, let us examine a little into the proof *you* give of the existence of *bacteria*. "Herr Professor" told you he "saw them," and, alas, for poor human credulity, that so often bolts down error upon its mere assertion, especially if of foreign origin, but scorns truth, however strongly sustained by proof, you questioned no part of his assertion.

Nobody disputes, or, at least, I do not and never have, that the "Herr Professor," and other microscopists, have seen in the exudations of diphtheria certain objects, as they assert; indeed, I maintain that they have seen them, and thus far reported truthfully upon what they saw; but at the same time I deny that these objects are vegetable parasites, as claimed; but, instead of that, I re-assert, that their microscocci, or spherical bacteria of diphtheria, are simply molecular granules of fibrin; that their rod-like bacteria are "fine, thread-like prolongations" of fibrin; and that their spiral bacteria are the same, or similar, threads of fibrin, contracted into spiral form, under their firmer organization.

These three forms of fibrin are certainly present, and in almost infinite numbers, in and about every diphtheritic membrane, and are utterly indistinguishable in form, feature or organization, from anything the microscopists have yet told us of the three classified forms of bacteria. And, furthermore, as these fibrinous bodies occupy the same positions, and demean themselves in precisely the same manner that we are told the bacteria do, I maintain that the burden of proof lies wholly with the advocates of the latter, to show us wherein the two are unlike and clearly distinguishable from each other, or we are no longer bound to believe in their claims. And especially of so unnatural a claim as that such vast hordes of *vegetable* organisms, which are so utterly foreign to animal life, are present in every case of diphtheria, and in possession of the blood, as well as of the tissues wherever exuded.

Fibrin being part of the blood, it is not foreign to it, or to the system at large, though it may be, and is, to the mucous membrane of the fauces or other parts, when poured out there in quantity from its *excess* in the blood; hence, I repeat, this is a *natural* and not forced accounting for all the results upon the most simple basis, in contradistinction to all points in the bacteria theory. An unnatural theory must be sustained by the most absolute proof, or we are not bound to receive it.

As to your remark: "It is more than likely he [the 'Herr Professor'] was quite familiar with the modes and action of fibrin while gathering into a clot, as it is some time since this ceased to be a novelty;" the sufficient answer is, that he or no other investigator of, or writer upon, diphtheria, ever applied that *fact*, of the peculiar action of fibrin in fibrillating, to the solution of the mystery of the exudations of diphtheria, until your humble servant did it; and it was begging the question, as well as a weak point for you to bring up in your criticism.

As to what you say of my assurances, "that there is not the slightest proof to show that these bodies are vegetable parasites," let us take a hasty glance. Three-fourths, or more, of all that has been written in the English language upon bacteria is based, more or less directly, upon what Oertel had written upon the subject. To his authority almost constant reference is made to sustain the doctrine. And it is a fair presumption that the leading advocate of a theory would give the best proof there was to establish it. Well, in the pamphlet you criticise, I quoted from Oertel as follows:

"The vegetable organisms which have been observed in the diphtheritic membranes of the fauces and air-passages, as well as in other products of the disease, belong to a group which comprises forms of such exceeding minuteness—for they stand upon the *very borders of the visible*—that, as yet, we possess *only the most unsatisfactory knowledge of their nature and organization.*" (The italics mine.)

And yet you did me the injustice, and yourself the discredit, as in other instances, of taking no notice of this whatever, any more than though it never had existence, and held me alone responsible for the assertion that there was no *satisfactory* proof as to these "organisms" being vegetable parasites. Or do you regard the positive assertion by Oertel, that given minute bodies which "stand upon the very borders of the visible," and that "only the most *unsatisfactory* knowledge of their nature and organization" is possessed, as good proof that they are whatever he or others may claim them to be?

Moreover, doctor, do you not see in this criticism, written so ostensibly to condemn all pathological investigations by our school, as well as to condemn my views, you have placed yourself in the unenviable position of an earnest advocate of a false pathology, and a most perniciously false pathology at that? You, a defender of, and apologist for, an allopathic and mongrel pathology of diphtheria, that is reeking through and through with fallacies, and which has led to much worse treatment than could otherwise have been thought of! I am astonished.

There is something, also, to be said of your laboring so hard through several pages to bring into ridicule my confidence in, and assertions of, nature's conservative efforts in the preservation of human life. Instead of your now enjoying a ripe old age, where would you be—where would any of us be—but for nature's incessant watchful care over us?

As thorough a student of Hahnemann as you have been, and for which I have honored, and still honor you, far more than you know, do you not know that when nature "steps in too far," of which you would make so much, she has the almost constant and unremitting efforts of the medical profession through hundreds of years in thwarting her beneficent purposes, to blame for the result?

For four hundred years, through every generation, and in almost every case, have doctors striven to thwart nature in her kindly efforts to force and keep syphilis to the surface, that she might thereby lessen its dangers and avert the horrors of the secondary and fatal internal disease; for thousands of years has she struggled in the same way and incessantly against doctors suppressing psora and many other forms of disease; and yet you, of all men, are now found ridiculing her because she sometimes, or quite often, if you please, fails to succeed against such tremendous odds, and "steps in too far," that "she, seemingly, does not know when to stop," "she means well, but don't know." What! that nature which, among so many other good deeds, "steps in" so often and arrests so terrible a disease as tuberculosis in the consumptive pregnant mother, that her offspring may be saved, "steps too far," does she, and don't know what she is about?

Ah, doctor, *you* "stepped in too far" when you penned those words, and "did not know," and the quicker you do know, and retrace those steps, the better it will be for your reputation as a medical philosopher. In every failure of nature to protect us, or our vital organs, from destruction or harm, she can point to hundreds of efforts through thousands of years, upon the part of our ancestors or their physicians, at forcing in upon delicate parts or organs some more or less deadly morbid poison, to be generally transmitted to posterity, like scrofula, syphilis, etc., and greatly weaken the forces of life in nearly all; and this is the full purport and meaning of all of Hahnemann's teachings and warnings upon the subject. I have myself done not a little investigating in this direction, and have found his warnings more than verified.

In ridiculing the idea that the pouring out of the excess of fibrin from the blood upon the tonsils, etc., and its there organizing into a membrane is, in any way, a conservative or beneficent act on the part of nature, did it not occur to you, could you not appreciate, that the question involved was one of *relative* danger; and that this exuded fibrin is far less dangerous when deposited upon any surface like that of the fauces, than if retained within those much more vital organs, the heart and arteries; that the continued retention within these, of all the fibrin exuded to make a large membrane would certainly kill in *every* case; whereas, its expulsion and formation of the membrane permits of the recovery of a third to a half or more of all cases, even under the worst forms of treatment, and of ninety-five hundredths, or more, of all cases, under the best homœopathic treatment? And could you not see that even in those terrible cases where fibrin is exuded into the larynx or bronchi, which I did not speak of, but you did to excite still more ridicule, the *danger is less*, and *more time is given to counteract it*, than though the same fibrin clotted in the heart or pulmonary artery?

If you ever had one of those cases where the membrane formed in the larynx, or bronchi, did you not think it would have been better and a beneficent act, had it all been organized in the fauces instead? Or, if you ever have a case of sudden death from thrombosis, will you not wish that the excess of fibrin had been thrown out of the blood, even if it was into the bronchi, and thus given you that little chance of cure, instead of no chance, but certain death?

And still again, could you not appreciate the *fact* that all the cases you have yourself cured, and all the two hundred and forty cases which you report with such evident pride, and I read of with much pleasure, as having every one been cured by two other physicians, that all of these and all your own cases, I repeat, where there was anything like a large amount of membrane formed, would have surely died, had that fibrin been retained in the circulation?

I did not say there was no danger from the membranes of diphtheria, or that the greater the membrane, the less the danger, as you would evidently have it inferred I did. On the contrary, I said: "Therefore, let the membranes of diphtheria, *although they are in themselves often so serious*, be henceforth looked upon in their true light as the work of the conservative efforts of nature to *avoid*," what? "Thrombosis and embolism, and a much more certainly fatal issue." And here again you did me a gross injustice in suppressing this paragraph, and then holding me just as responsible as though it had never been uttered.

And now, in conclusion of this point, and at the expense of some repetition, I repeat with more emphasis than ever before, that, given the excess of fibrin in the blood in diphtheria, it is a conservative and beneficent act of nature, and *far less dangerous*, to throw it out into the fauces, than into the larynx and bronchi; and although so often fatal in the latter position, it is nevertheless not so certainly fatal there, as when it coagulates in the heart or large arteries.

As to your making so light of thrombosis and embolism, and questioning their occurrence in any, or but very few cases, my comment is: I fear your evident enmity to pathology has betrayed you into a great neglect of pathological reading. If you will now please examine "Ziemsen's Cyclopædia of Medicine," you will find in the index of two-thirds, or more, of all its seventeen volumes, references to thrombosis, or embolism, or both, and in various diseases where fibrin is in excess in the blood, besides diphtheria; while a careful reading of those volumes will furnish you illustrations and descriptions of the great importance of this danger in several other diseases as well as in diphtheria. So, you see, doctor, here we have something like a lion, after all; or, something more than "only Snug, the joiner."

Now turn, also, to Jacobi's "Treatise on Diphtheria," page 114, and read this of seventeen *post-mortems* by Reimer: "In the *heart*, particularly the right, numerous thrombi in various stages of development were found;" that there were "emboli of the liver in three" cases, and "emboli of the spleen in five" cases. And this on page 115: "Bouchut and Labadie-Lagrave, out of fifteen cases of diphtheria, met with a plastic endocarditis in fourteen, which became the source of emboli;" also, "superficial thrombi of the small veins of the heart, subcutaneous connective tissue, pia mater, brain and liver." (I do not make statements, doctor, unless I know something of the facts of which I am speaking.)

From all this, then, and when you reflect that plastic endocarditis, and some other fatal complications of the heart besides this and thrombi, as well as of other vital organs, result from the excess of fibrin not being all expelled from the blood, you will be able to realize something of the *fact* that it is better—that it is even *beneficent*

—that the said fibrin should be cast out therefrom upon some surface, even though it may cause some, or very great, but still less, danger there, than when all retained.

And please permit me to say further, you cannot know the great, the towering superiority of your own, or others' pure homœopathic treatment of diphtheria, in saving all, or nearly all cases, unless you take into consideration how patients die, and what post-mortems reveal in those who die of this disease under other methods of treatment. You greatly lower your own standard when you seize upon the weapon of ridicule to cast distrust upon the real dangers that autopsies in great numbers have shown must environ every serious case of this terrible disease.

Do not, I beseech you, belittle your own great successes by trying to make out that the dangers of diphtheria are far less than all thorough pathologists well know them to be in every marked case of the disease. It is in this, and similar ways, that homœopathy is often greatly injured by some of our best men.

And here is as good a place as any to laugh a little at you for taking me to task on treatment, when, had you read my book, as you ought, if you were going to speak at all upon the subject, you would have found that part of it as strictly homœopathic, or more so, than any you ever proclaimed.

There remains much more to be said upon the points here considered, as well as upon many others, in connection with diphtheria, which the limits of a journal article will not allow of being discussed therein; therefore, in conclusion, I will make you this proposition: If you will secure the place for discussion, and arrange that it shall be entirely fair to all concerned, you may associate with you three, five, or more, other physicians, and I will meet you all and discuss every question in connection with diphtheria, you may desire to raise. The only conditions I will make, will be, that I shall be given an hour, or hour and a half, at first, to lay before you my views upon various branches of the subject, then be given proper time at subsequent meetings, and all within a few days, to answer every objection I can, that may be thought valid against me.

Now, this offer is made in good faith, and not in a boastful spirit, nor in anticipation of an easy triumph; but I candidly believe that if we can come together in the right spirit, and consider the whole subject as we ought, we may, perhaps, be able to settle upon a true foundation for the pathology and ætiology of diphtheria for all time, and show to the world what must and what must not be done in its treatment, to save the greatest proportion of cases.

